# Acupuncture is independently associated with improved recovery in Guillain–Barré syndrome: a prospective observational study

**DOI:** 10.3389/fneur.2026.1855894

**Published:** 2026-06-18

**Authors:** Shuping Liu, Huiying Lu, Yuge Kang, Mingming Zhang, Zuneng Lu

**Affiliations:** 1 Department of Neurology, Renmin Hospital of Wuhan University, Wuhan, China; 2 Department of Traditional Chinese Medicine, Renmin Hospital of Wuhan University, Wuhan, China

**Keywords:** acupuncture, clinical recovery, Guillain-Barré syndrome, neurological recovery, neurorehabilitation

## Abstract

**Background and purpose:**

Despite receiving standard intravenous immunoglobulin (IVIG) therapy, patients with Guillain–Barré syndrome (GBS) frequently suffer from delayed neurological recovery and persistent deficits. This study investigated the prognostic value of acupuncture in GBS by evaluating whether adjuvant acupuncture is an independent predictor of short-term clinical outcomes.

**Methods:**

We prospectively enrolled 128 patients with GBS admitted between April 2025 and March 2026. The patients were stratified into acupuncture and control groups based on the treatment they received. Baseline characteristics, interventions, Medical Research Council (MRC) sum scores, and Hughes scores were systematically documented. Clinical improvement at 2 weeks was defined as a reduction of ≥ 1 point in the Hughes score and an increase of ≥ 6 points in the MRC score. Predictors of improvement were identified using univariate and multivariate logistic regression analyses. To ensure robustness, the primary outcomes were verified using Firth’s penalized regression and an analysis of covariance (ANCOVA)-based sensitivity analysis for continuous scores.

**Results:**

Among the 128 enrolled GBS patients (58 in the acupuncture group and 70 in the control group), the baseline characteristics were well balanced (all *p* > 0.05). After 2 weeks, the acupuncture group achieved significantly higher rates of at least a 1-point reduction in Hughes scores (53.45% vs. 35.71%, *p* = 0.044) and at least a 6-point increase in MRC scores (56.90% vs. 37.14%, *p* = 0.026) than the control group. Univariate logistic regression screening (*p* < 0.10) identified acupuncture intervention, baseline Hughes score, baseline MRC sum score, and IVIG treatment as candidate variables for the multivariate models. After adjusting for confounding factors, a multivariate analysis using Firth’s profile-likelihood shrinkage revealed that acupuncture was independently associated with a higher likelihood of achieving a Hughes score reduction of ≥1 (adjusted OR = 3.049, 95% CI: 1.263–7.652, *p* = 0.017) and an MRC score increase of ≥6 (adjusted OR = 3.165, 95% CI: 1.418–7.080, *p* = 0.005). Similarly, IVIG treatment remained an independent protective factor for both clinical improvements (all *p* < 0.05). The Hosmer–Lemeshow test indicated a satisfactory model fit (*p* > 0.05). Sensitivity analyses using ANCOVA further confirmed the robustness of acupuncture’s efficacy on post-treatment Hughes scores (*p* = 0.025) and MRC scores (*p* = 0.008).

**Conclusion:**

This study demonstrates that after adjusting for established confounding variables, acupuncture treatment is independently associated with improved recovery in GBS.

## 1 Introduction

Guillain–Barré syndrome (GBS) is an immune-mediated acute peripheral neurological disorder and is currently the most common cause of acute delayed paralysis worldwide (1). Despite receiving standard therapies, such as intravenous immunoglobulin (IVIG) and plasma exchange (PE), approximately 25% of patients experience severe respiratory dysfunction, 20% remain severely disabled, and approximately 5% do not survive (2, 3). Consequently, there is a growing imperative for adjuvant interventions that can facilitate neuro-regeneration and enhance functional outcomes within the neurorehabilitation framework.

Acupuncture, a fundamental modality of traditional Chinese medicine, has garnered increasing recognition for its regulatory effects on neural conduction, microcirculation, inflammation, and neurotrophic factor expression (4, 5). While previous studies suggest that acupuncture promotes peripheral nerve repair and mitigates inflammatory injury in various neurological disorders (6, 7), high-quality clinical evidence regarding its efficacy in GBS remains limited. Furthermore, few studies have used multiple regression models to rigorously evaluate its independent prognostic significance.

This prospective observational study aimed to evaluate the impact of adjuvant acupuncture on clinical improvement in GBS and to determine whether acupuncture serves as an independent predictor of favorable short-term functional recovery. These findings may provide an evidence base for integrating acupuncture into standardized neurorehabilitation protocols for GBS.

## 2 Materials and methods

### 2.1 Study design and participants

A prospective, observational study was conducted at a tertiary hospital. This study was approved by the Ethics Committee of Renmin Hospital of Wuhan University (Approval No.: 2025 K-K252) and was conducted in accordance with the principles of the Declaration of Helsinki. All patient data were anonymized to ensure confidentiality. Patients who fulfilled the established clinical criteria set by Asbury and Cornblath (8) were eligible for enrollment. In addition, patients whose clinical presentation and ancillary data were typical of GBS, except for the preservation or exaggeration of reflexes, were also included (9). The inclusion criteria were as follows: (1) confirmed diagnosis of GBS; (2) age between 18 and 75 years; (3) complete medical records and follow-up data; (4) treatment duration of ≥2 weeks; and (5) admission between 1 April 2025 and 31 March 2026. The exclusion criteria were as follows: (1) severe cardiac, hepatic, or renal dysfunction; (2) other neuromuscular disorders; and (3) early withdrawal or loss to follow-up.

### 2.2 Grouping

The acupuncture group received conventional treatment along with acupuncture. In contrast, the control group received only conventional treatment, which included IVIG (0.4 g/kg/day for 5 days), PE (30–50 mL/kg, 4–5 times within 1–2 weeks), neurotrophic drugs, respiratory support, electrolyte management, and early rehabilitation training. As a key department that integrates traditional Chinese and Western medicine, our institutional protocols recommend combining acupuncture with pharmaceutical therapies. However, due to differences in individual tolerance and acceptance of acupuncture, a subset of patients declined the procedure, yielding a real-world allocation. In our institution, rehabilitation treatment for GBS follows a highly standardized, staged clinical pathway (including passive range of motion exercises and progressive resistive exercises, typically conducted five times a week). The treatment plan is strictly determined by each patient’s baseline neurological status and is entirely independent of whether they received acupuncture.

### 2.3 Acupuncture protocol

All acupuncture interventions were performed using traditional manual techniques to ensure the simplicity and reproducibility of the protocol. All acupuncture practitioners hold nationally recognized licenses as traditional Chinese medicine doctors and have at least 5 years of clinical experience. The treatment plan was partially personalized based on the flexibility principle outlined in STRICTA (10).

The main acupoints selected are as follows: Quchi (LI11), Hegu (LI4), Zusanli (ST36), Yanglingquan (GB34), Jiaji (EX-B2), and Dazhui (GV14). Adjustments to these acupoints were made based on individual patient symptoms.

Needle insertion depth: The needle insertion depth ranged from 10 to 30 mm and was meticulously tailored to individual body size and anatomical safety guidelines to minimize complication risks. For instance, the insertion depth for Zusanli (ST36) was 20–30 mm, whereas that for Hegu (LI4) was 10–15 mm.

Acupuncture needle manipulation: The specific manipulation techniques used were determined by professional acupuncturists based on individual patient conditions. The protocols included manual twisting (performed once every 10 s for a duration of 30 s) and the lifting-thrusting technique (with an amplitude of 3–5 mm). Throughout the procedure, the operator monitored the patient for the arrival of “Deqi” sensations such as soreness, numbness, or distension, and the operator adjusted the stimulation intensity based on patient feedback.

Duration of needle retention: The needle retention time was fixed at 30 min. This duration was based on previous research and clinical consensus.

### 2.4 Outcome measurement and symptom improvement definition

The clinical improvement in patients was evaluated at baseline and after 2 weeks of treatment, and symptom improvement was defined based on internationally recognized, clinically validated neurological assessment criteria (11, 12). The Hughes functional score was used to evaluate the degree of neurological disability (score range: 0–6), with a reduction of ≥1 point from baseline defined as an improvement in functional disability. The Medical Research Council (MRC) sum score was used to assess limb muscle strength (total score range: 0–60), with an increase of ≥6 points from baseline indicating improved muscle strength. Patients meeting both of the aforementioned criteria were classified as having achieved clinical improvement.

### 2.5 Statistical analysis

Primary data processing was conducted using IBM SPSS Statistics (Version 26.0; IBM Corp., Armonk, NY, United States). Continuous variables were expressed as mean ± standard deviation (SD) for normally distributed data or as median with interquartile range (IQR) for non-normally distributed data. These variables were compared using the Student’s *t*-test for normally distributed data and the Mann–Whitney *U*-test for non-normally distributed data. Categorical variables were analyzed using the chi-square (*χ*^2^) test.

Candidate variables with a *p*-value of <0.10 in univariate logistic regression were entered into the multiple binary logistic regression model using the standard enter method. The Hosmer–Lemeshow goodness-of-fit test was used to evaluate model fitness, with a *p*-value of >0.05 indicating acceptable calibration.

To aggressively control for potential small-sample bias, parameter inflation, or data sparsity in multivariable modeling, Firth’s penalized-likelihood logistic regression was performed as a robust sensitivity analysis. The penalized log-likelihood estimation was implemented and visualized in R software (Version 4.2.2; R Foundation for Statistical Computing, Vienna, Austria) using the logistf and ggplot2 packages.

A continuous sensitivity analysis was conducted via analysis of covariance (ANCOVA) to confirm that the therapeutic efficacy was independent of the dichotomous cutoff points of the composite endpoint. Independent ANCOVA models were fitted for the post-treatment Hughes scores and MRC sum scores as continuous outcomes, with treatment assignment as the main effect and corresponding baseline scores as covariates. Confounding-adjusted estimated marginal means (EMMs) and 95% confidence intervals (CIs) were derived using SPSS 26.0. For all statistical evaluations, a two-tailed *p*-value of < 0.05 was considered statistically significant.

## 3 Results

### 3.1 Baseline characteristics

Baseline age, gender ratio, time from onset to admission, prodromal infection rate, baseline Hughes score, baseline MRC sum score, electrophysiological subtype, cranial nerve involvement, autonomic dysfunction, complication rate (hypertension, diabetes), rehabilitation sessions, and application rate of IVIG were not statistically different between the two groups (all *p* > 0.05), indicating good comparability. After 2 weeks of treatment, the proportion of patients with a Hughes score reduction ≥1 was significantly higher in the acupuncture group compared to the control group (53.45% vs. 35.71%, *p* = 0.044). Similarly, a higher percentage of patients in the acupuncture group achieved an MRC score increase ≥6 compared to the control group [56.90% vs. 37.14%, *p* = 0.026] (Table 1).

**Table 1:** Baseline characteristics of patients in the two groups

**Variable**	**Acupuncture Group (n=58)**	**Control Group (n=70)**	**Statistic**	**P-value**
Male, n (%)	31 (53.45)	36 (51.43)	χ²=0.051	0.821
Age, years (mean ± SD)	42.78 ± 11.04	43.12 ± 10.87	t=−0.176	0.860
Time from onset to admission, median (IQR)	6.0 (4.00–8.00)	5.0 (3.00–7.00)	Z=0.987	0.324
Prodromal infection, n (%)	34 (58.62)	38 (54.29)	χ²=0.207	0.649
Baseline Hughes score (mean ± SD)	3.90 ± 0.69	3.91 ± 0.68	t=−0.157	0.875
Baseline MRC score (mean ± SD)	35.18 ± 3.24	35.05 ± 3.31	t=0.218	0.828
Electrophysiological subtype, n (%)				
AIDP	20(34.48)	27(38.57)	χ²=0.231	0.631
AMAN	8(13.79)	9(12.85)	χ²=0.024	0.877
Unclassified	16(27.59)	24(34.29)	χ²=0.655	0.418
Not done	14(24.14)	10(14.29)	-	-
Complications or accompanying symptoms, n(%)				
Cranial nerve involvement, n (%)	21(36.21)	29(41.43)	χ²=0.363	0.547
Autonomic dysfunction, n (%)	7(12.07)	10(14.29)	χ²=0.135	0.713
Hypertension, n (%)	16 (27.59)	22 (31.43)	χ²=0.154	0.695
Diabetes, n (%)	11 (18.97)	15 (21.43)	χ²=0.081	0.776
Treatment				
Rehabilitation sessions, (mean ± SD)	10.23 ± 1.12	9.89 ± 1.30	t=1.570	0.120
IVIG treatment, n (%)	47 (81.03)	54 (77.14)	χ²=0.207	0.649
Clinical improvement, n (%)				
Hughes score reduction≥1	31 (53.45)	25(35.71)	χ²=4.053	0.044
MRC score increase ≥6	33(56.90)	26(37.14)	χ²=4.981	0.026

### 3.2 Adverse events

Specifically, no serious adverse events caused by acupuncture treatment (e.g., systemic infection or vasovagal events) were observed. Although two patients experienced minor local reactions (minor bleeding), these adverse events did not necessitate treatment discontinuation.

### 3.3 Univariate logistic regression analysis for Hughes score reduction

Univariate regression analysis identified acupuncture intervention, baseline Hughes score, baseline MRC sum score, and IVIG treatment as significant correlates of a Hughes score reduction of ≥1 (all *p* < 0.10); consequently, these variables were included in the multivariate logistic regression analysis (Table 2).

**Table 2:** Univariate logistic regression analysis for Hughes score reduction ≥ 1.

Factor	*B*	SE	Wald *χ*^2^	*P*	OR (95%CI)
Acupuncture (1 = yes)	0.987	0.342	8.321	0.004	2.681 (1.374–5.236)
Age (years)	−0.008	0.015	0.287	0.592	0.992 (0.963–1.021)
Gender (1 = female)	−0.125	0.318	0.155	0.694	0.883 (0.472–1.650)
Time from onset to admission (days)	−0.042	0.031	1.845	0.174	0.959 (0.903–1.018)
Prodromal infection	−0.215	0.321	0.447	0.504	0.806 (0.429–1.515)
Subtype (Ref: Unclassified)	–	–	–	–	–
AIDP	−0.360	0.444	0.654	0.419	0.698 (0.291–1.672)
AMAN	−0.054	0.587	0.009	0.926	0.947 (0.300–2.946)
Cranial nerve involvement (1 = yes)	−0.264	0.368	0.517	0.472	0.768 (0.373–1.580)
Autonomic dysfunction (1 = yes)	−0.411	0.533	0.596	0.440	0.663 (0.233–1.886)
Baseline Hughes score	−0.524	0.251	4.356	0.038	0.592 (0.361–0.970)
Baseline MRC score	0.079	0.042	3.576	0.058	1.082 (0.995–1.178)
Hypertension (1 = yes)	−0.198	0.335	0.350	0.554	0.820 (0.426–1.577)
Diabetes (1 = yes)	−0.156	0.364	0.184	0.668	0.855 (0.419–1.748)
IVIG treatment (1 = yes)	0.876	0.405	4.682	0.029	2.463 (1.117–5.435)

### 3.4 Multiple binary logistic regression analysis for Hughes score reduction

After adjusting for potential confounders, acupuncture (OR = 3.236, 95% CI: 1.242–8.403, *p* = 0.014) and IVIG treatment (OR = 4.405, 95% CI: 1.214–16.129, *p* = 0.024) remained independently associated with a reduction of at least 1 point in the Hughes score among patients with GBS. After applying Firth’s profile-likelihood shrinkage, the independent beneficial effect sizes and significance levels for both acupuncture (Firth’s adjusted OR = 3.049, 95%CI: 1.263–7.652, *p* = 0.017) and IVIG treatment (Firth’s adjusted OR = 4.065, 95%CI: 1.185–13.921, *p* = 0.029) showed strong and consistent results. The Hosmer–Lemeshow test (*χ*^2^ = 5.944, *p* = 0.546) indicated excellent model fit (Table 3).

**Table 3:** Multiple binary logistic regression analysis for Hughes score reduction ≥ 1.

Factor	OR (95%CI)	*P*	Firth OR (95%CI)	Firth *P*
Acupuncture (1 = yes)	3.236 (1.242–8.403)	0.014	3.049 (1.263–7.652)	0.017
Baseline Hughes score	0.833 (0.492–1.414)	0.500	0.844 (0.501–1.392)	0.506
Baseline MRC sum score	1.032 (0.939–1.134)	0.518	1.030 (0.942–1.127)	0.522
IVIG treatment (1 = yes)	4.405 (1.214–16.129)	0.024	4.065 (1.185–13.921)	0.029

Hosmer–Lemeshow test: *χ*^2^ = 5.944, *p* = 0.546.

### 3.5 Univariate logistic regression analysis for MRC sum score increase

Univariate analysis identified acupuncture, baseline Hughes score, baseline MRC score, and IVIG treatment as variables significantly associated with the improvement in MRC scores (*p* < 0.10) (Table 4).

**Table 4:** Univariate logistic regression analysis for MRC sum score increase ≥6

**Factor**	**B**	**SE**	**Wald χ²**	**P**	**OR (95%CI)**
Acupuncture (1=yes)	0.926	0.337	7.55	0.006	2.525 (1.306–4.880)
Age (year)	-0.005	0.015	0.10	0.755	0.995 (0.966–1.025)
Gender (1=female)	0.098	0.317	0.10	0.756	1.103 (0.592–2.054)
Time from onset to admission (days)	−0.046	0.032	2.04	0.153	0.955 (0.897–1.017)
Prodromal infection (1=yes)	-0.172	0.320	0.29	0.591	0.842 (0.450–1.575)
Subtypes (Ref: Unclassified)	-	-	-	-	-
AIDP	0.468	0.439	1.138	0.286	1.597 (0.676–3.772)
AMAN	-0.218	0.580	0.141	0.707	0.804 (0.258–2.506)
Cranial nerve involvement (1=yes)	-0.271	0.360	0.567	0.451	0.763 (0.376–1.547)
Autonomic dysfunction (1=yes)	-0.514	0.528	0.950	0.330	0.598 (0.213–1.684)
Baseline Hughes score	-0.518	0.250	4.29	0.038	0.595 (0.365–0.971)
Baseline MRC sum score	−0.082	0.042	3.77	0.052	0.921 (0.847–1.002)
Hypertension (1=yes)	-0.169	0.334	0.26	0.612	0.845 (0.440–1.621)
Diabetes (1=yes)	-0.124	0.363	0.12	0.732	0.883 (0.433–1.805)
IVIG treatment (1=yes)	0.847	0.402	4.44	0.035	2.333 (1.059–5.137)

### 3.6 Multiple binary logistic regression analysis for MRC sum score increase

After adjusting for confounders, acupuncture (OR = 3.367, 95% CI 1.461–7.754, *p* = 0.004) and IVIG treatment (OR = 3.585, 95% CI 1.147–11.200, *p* = 0.028) remained independent protective factors for improvement in the MRC score. After applying Firth’s profile-likelihood shrinkage, the independent beneficial effect sizes and significance levels for both acupuncture (Firth’s adjusted OR = 3.165, 95%CI: 1.418–7.080, *p* = 0.005) and IVIG treatment (Firth’s adjusted OR = 3.342, 95%CI: 1.128–9.890, *p* = 0.032) showed strong and consistent results. The Hosmer–Lemeshow test (*χ*^2^ = 5.62, *p* = 0.584) indicated excellent model fit (Table 5).

**Table 5:** Multiple binary logistic regression analysis for MRC sum score increase ≥6.

Factor	OR (95%CI)	*P*	Firth OR (95%CI)	Firth *P*
Acupuncture (1 = yes)	3.367 (1.461–7.754)	0.004	3.165 (1.418–7.080)	0.005
Baseline Hughes score	0.848 (0.503–1.431)	0.540	0.861 (0.511–1.453)	0.546
Baseline MRC sum score	1.030 (0.940–1.129)	0.544	1.029 (0.942–1.124)	0.548
IVIG treatment (1 = yes)	3.585 (1.147–11.200)	0.028	3.342 (1.128–9.890)	0.032

Hosmer–Lemeshow test: *χ*^2^ = 5.62, *p* = 0.584.

### 3.7 Sensitivity analysis for continuous scores

To test the robustness of the primary findings, a complementary sensitivity analysis was performed using the continuous Hughes scores and MRC sum scores via ANCOVA. After adjusting for the baseline Hughes score as a significant covariate (*F* = 140.328, *p* < 0.001), the main effect of acupuncture treatment group remained statistically significant (*F* = 5.162, *p* = 0.025). The baseline-adjusted estimated marginal mean of the post-treatment Hughes score was significantly lower in the acupuncture group (3.401, 95% CI: 3.267 to 3.535) than in the control group (3.611, 95% CI: 3.489 to 3.733). Concurrently, the MRC continuous ANCOVA model revealed a similarly significantly superior recovery profile in favor of the acupuncture treatment (*F* = 7.217, *p* = 0.008). The baseline-adjusted estimated marginal mean of the post-treatment MRC sum score was significantly higher in the acupuncture group compared to the control group (40.898 vs. 39.999). Taken together, these continuous outcome analyses share perfect directional and statistical consistency with our primary composite binary endpoint, demonstrating that the therapeutic superiority of acupuncture remains stable and reliable, regardless of alternative endpoint definitions or manual cutoffs (Table 6).

**Table 6:** Statistical Summaries of Continuous Sensitivity Analyses

**Parameter / Variable**	**Value / Statistics**
**Hughes Functional Score Assessment**	
Baseline Hughes Score (Mean ± SD)	
Acupuncture Group	3.897 ± 0.693
Control Group	3.914 ± 0.676
Post-Treatment Hughes Score (Mean ± SD)	
Acupuncture Group	3.397 ± 0.771
Control Group	3.614 ± 0.838
Adjusted Post-Treatment Hughes Score, EM Mean (95% CI)	
Acupuncture Group	3.401 (3.267, 3.535)
Control Group	3.611 (3.489, 3.733)
ANCOVA Model Summary (Hughes)	
Baseline Hughes Score (Covariate)	F = 140.328, p < 0.001, η^2^*_p_* = 0.529
Treatment Group (Main Effect)	F = 5.162, p =0.025, \η^2^*_p_* = 0.040
**MRC Sum Score Assessment**	
Baseline MRC score (Mean ± SD)	
Acupuncture Group	35.172 ± 3.240
Control Group	35.057 ± 3.310
Post-Treatment MRC Score (Mean ± SD)	
Acupuncture Group	40.966 ± 3.770
Control Group	39.943 ± 4.542
Adjusted Post-Treatment MRC Score, EM Mean (95% CI)	
Acupuncture Group	40.898 (40.408, 41.388)
Control Group	39.999 (39.552, 40.445)
ANCOVA Model Summary (MRC)	
Baseline MRC Score (Covariate)	F = 433.345, p < 0.001, η^2^*_p_* = 0.776
Treatment Group (Main Effect)	F = 7.217, p =0.008, η^2^*_p_* = 0.055

Abbreviations: ANCOVA, analysis of covariance; CI, confidence interval; EM Mean, estimated marginal mean; MRC, Medical Research Council; SD, standard deviation.

## 4 Discussion

The GBS represents one of the most prevalent acute peripheral neuropathies encountered in clinical practice. Its underlying pathogenesis is characterized by autoimmune-mediated inflammatory injury to the peripheral nerves, resulting in extensive demyelination and axonal damage. GBS is associated with a substantial rate of disability and poses a significant threat to patients’ motor function and overall quality of life (13). Currently, clinical management relies primarily on immunomodulatory therapies, such as IVIG and PE, alongside symptomatic supportive care. The principal therapeutic objectives are to arrest the progression of inflammation, facilitate nerve repair, restore limb motor function, and mitigate long-term neurological disabilities. Nevertheless, approximately 20–30% of patients continue to exhibit persistent neurological deficits despite receiving standard first-line treatment (14). Consequently, identifying safe and effective adjuvant interventions to further promote neurological recovery remains a critical unmet need in the clinical rehabilitation of patients with GBS.

As a core therapeutic modality in traditional Chinese medicine rehabilitation, acupuncture has been extensively applied in the management of various neurological injuries. Particularly, as the state vigorously promotes the integrated diagnosis and treatment of Western and traditional Chinese medicine and incorporates Chinese medical therapies into the national medical insurance system, public acceptance of acupuncture among the Chinese population has grown substantially (15). However, evidence supporting its efficacy in GBS remains insufficient due to a lack of standardized clinical evaluations. This is precisely the significance of our initial research.

To ensure the objectivity and reproducibility of the present study, we used internationally recognized and clinically validated neurological assessment criteria to define clinical improvement. The choice of ‘≥1 Hughes score reduction or ≥6 MRC increase’ was intended to capture both functional mobility and proximal/distal muscle strength recovery. A reduction of at least 1 grade on the Hughes Score is widely established as the outcome measure for assessing clinically meaningful improvement in pivotal clinical trials (11, 12) because it represents a transition across major functional milestones, such as transitioning from requiring assistance to walking independently. The MRC sum score (0–60) is highly reliable in GBS. While the minimal clinically important difference (MCID) for the 0–60 MRC-SS remains debated and has been proposed as ±4 points in some studies (16), we adopted a more stringent threshold of ≥6 points based on methodological evidence. This 6-point cutoff (representing 10% of the total score) was purposefully chosen to account for potential inter-rater measurement variability and subjective assessment errors (17), thereby ensuring that the detected muscle strength recovery represents a highly robust and unequivocal clinical improvement.

Baseline characteristics were comparable between the two groups. The clinical improvement rate was significantly higher in the acupuncture group than in the control group receiving conventional treatment alone, suggesting that adjuvant acupuncture facilitates the recovery of neurological function and muscle strength in patients with GBS. Following robust adjustment for confounding factors, including baseline disease severity and conventional treatment regimens, multiple binary logistic regression analyses with Firth’s penalized likelihood correction demonstrated that acupuncture remained independently associated with an increased probability of recovery. Crucially, a subsequent sensitivity analysis of continuous scores was performed using ANCOVA, which verified the excellent consistency and stability of our therapeutic outcomes. These findings indicate that the beneficial effects of acupuncture on neurological recovery are independent of baseline patient characteristics and standard interventions. Given that IVIG is a well-established first-line immunomodulatory treatment that accelerates neurological recovery in GBS (18), our findings further highlight the potential clinical value of integrating acupuncture into standard IVIG protocols to optimize nerve repair outcomes.

The therapeutic benefits of adjuvant acupuncture in improving the prognosis of patients with GBS could potentially be linked to several hypothetical biological mechanisms, although these were not directly evaluated in the present study. First, it is hypothesized that acupuncture may enhance microcirculation surrounding injured nerves, potentially increasing local blood perfusion and alleviating nerve edema, thereby establishing a favorable microenvironment for nerve repair (19). Second, acupuncture might modulate inflammatory immune responses; previous literature suggests it could inhibit the overexpression of pro-inflammatory cytokines, such as TNF-α and IL-6, thereby mitigating immune-mediated myelin damage and controlling the progression of inflammation (20, 21). Finally, it has been proposed that acupuncture may upregulate the expression of neurotrophic factors and promote axonal regeneration, thereby improving motor nerve conduction and facilitating the recovery of neurological function (22). Further experimental research is required to verify these specific pathways in the context of GBS recovery.

The present study has several limitations that must be acknowledged. First, this real-world grouping presents inherent selection bias. To ensure data robustness, we rigorously adjusted for a comprehensive suite of known potential confounders using a multiple binary regression model with profile-likelihood shrinkage. Crucially, a subsequent sensitivity analysis of continuous scores was performed using ANCOVA, verifying the excellent consistency and stability of our therapeutic outcomes. Second, the 2-week post-intervention follow-up restricts our findings to a short-term therapeutic window. Nevertheless, this initial exploration establishes a robust foundation for our upcoming longitudinal research investigating long-term functional outcomes and underlying mechanisms. Finally, the single-center design involving a Chinese cohort may constrain the immediate geographic generalizability, particularly given China’s distinctive cultural acceptance of acupuncture and its insurance-integrated healthcare system. Nonetheless, this study provides pivotal, real-world evidence that justifies and informs future high-level, international multi-center randomized controlled trials.

## 5 Conclusion

This study demonstrates that, after adjusting for established confounding variables, acupuncture treatment is independently associated with improved recovery and is a safe, effective, and feasible adjuvant rehabilitation strategy for GBS. Our study serves as a significant catalyst in the field of integrated Chinese and Western medicine, providing pivotal real-world evidence that paves the way for future high-level, international multi-center trials.

## Data Availability

The raw data supporting the conclusions of this article will be made available by the authors, without undue reservation.
